# Common framework mutations impact antibody interfacial dynamics and flexibility

**DOI:** 10.3389/fimmu.2023.1120582

**Published:** 2023-02-23

**Authors:** Emily R. Rhodes, Jonathan G. Faris, Brian M. Petersen, Kayla G. Sprenger

**Affiliations:** Department of Chemical & Biological Engineering, University of Colorado, Boulder, CO, United States

**Keywords:** antibodies, flexibility, framework mutations, molecular dynamics simulations (MD), antibody interfacial dynamics

## Abstract

**Introduction:**

With the flood of engineered antibodies, there is a heightened need to elucidate the structural features of antibodies that contribute to specificity, stability, and breadth. While antibody flexibility and interface angle have begun to be explored, design rules have yet to emerge, as their impact on the metrics above remains unclear. Furthermore, the purpose of framework mutations in mature antibodies is highly convoluted.

**Methods:**

To this end, a case study utilizing molecular dynamics simulations was undertaken to determine the impact framework mutations have on the VH-VL interface. We further sought to elucidate the governing mechanisms by which changes in the VH-VL interface angle impact structural elements of mature antibodies by looking at root mean squared deviations, root mean squared fluctuations, and solvent accessible surface area.

**Results and discussion:**

Overall, our results suggest framework mutations can significantly shift the distribution of VH-VL interface angles, which leads to local changes in antibody flexibility through local changes in the solvent accessible surface area. The data presented herein highlights the need to reject the dogma of static antibody crystal structures and exemplifies the dynamic nature of these proteins in solution. Findings from this work further demonstrate the importance of framework mutations on antibody structure and lay the foundation for establishing design principles to create antibodies with increased specificity, stability, and breadth.

## 1 Introduction

The design of antibodies (Abs) for a wide variety of purposes, including therapeutics (1, 2), biosensing (3), and separations, has exploded in the last few decades due in part to the ever-growing body of literature describing the many different features that can be tuned to engineer “successful” Abs (4). In particular, understanding the role structural rigidity plays in the binding affinity and specificity of Abs has long been a sought-after goal (5, 6), and continues to be a component of modern engineering efforts (7).

The genes which encode these marvels of our immune systems are a combination of V, D, J, and C genes that are stochastically cut and spliced together, and this recombination, in addition to somatic hypermutation, enables the incredible diversity of—and robust protection provided by—the adaptive immune system (8). As sequencing technology has become cheaper and more specific, many studies, databases, and techniques have been created to better understand the complex systems and pathways our immune system utilizes to protect us (9). However, as we continue to resolve more accurate and diverse Ab structures, we must seek to better understand the dynamic Ab response to a given antigen, mutation, etc. A deeper understanding of the structural dynamics of these Abs will enable us to design more efficacious therapies, sensors, and assays.

To grasp the importance of shedding the “static dogma” of Ab structures, we can look to two recently-published articles from the Liedl group (10, 11). Briefly, their work highlights the importance of the interface angle between the variable region of the immunoglobulin heavy chain (*V_H_*) and light chain (*V_L_*), and its impact on germline (GL) structure macrostates. We recently explored the impact of common framework mutations in baseline human Ab repertoires with the goal of understanding the effects of these mutations on the developability of monoclonal Abs (mAbs) (12). Here, we sought to elucidate new underlying features of Ab interfacial dynamics through a case study of these past simulations.

Abs evolve *in vivo* through a stochastic, Darwinian evolutionary process known as affinity maturation (13–15). Starting from a GL sequence, B cells undergo affinity maturation when exposed to a pathogen or vaccine. Their B cell receptors (BCRs)—which are later secreted as soluble Abs—undergo somatic hypermutation to develop high affinity and specificity for their cognate antigen. Canonically understood to be primarily driven by affinity-based selection criteria, there have been interesting studies which highlight the importance of flexibility in generating a robust Ab response (16). It remains puzzling why increases in Ab flexibility would be selected for during affinity maturation as they have associated enthalpic costs that increase the free energy of folding of the Ab, making it wholistically more unstable. Therefore, to continue being selected, the BCRs must acquire a competitive advantage by incorporating this flexibility.

If we adopt a more dynamic view of Ab structures, rather than a view based merely on static Ab structures (e.g., crystal structures), we believe the advantage could arise from an increased number of stable macrostates available in the Ab’s conformational ensemble, where macrostates are semi-stable states that the Ab structure visits (10, 11). Additional macrostates provide the antibodies with a larger conformational space to explore when encountering diverse antigens. The resulting increases in flexibility would enable rearrangement of the *V_H_-V_L_* interface and the complementarity-determining regions (CDRs) to suit the antigen being presented at a given time. Further studies characterizing these dynamic macrostates both *in silico* and *in vitro* are needed to deepen our understanding of the diversity of macrostates and the role these states play in Ab stability and breadth. Utilizing the conformational ensemble perspective to better inform Ab engineering efforts could result in more successful clinical trials for therapeutic Abs, more specific sensing technologies, and more effective vaccines against highly mutable pathogens like HIV (17–19), Malaria (20), and Influenza (21).

Current *in vitro* Ab engineering techniques (e.g., directed evolution), typically do not capture increases in Ab flexibility, but instead select primarily for mutations that increase the binding affinity, which generally rigidify the protein (22). Because a more rigid structure can more tightly bind to a single epitope, rigidification happens in nature as well, when Abs undergo somatic hypermutation (16). However, in nature, Abs may also incorporate flexibility during the maturation process when beneficial for Ab breadth (i.e., when Abs are exposed and need to be able to bind sufficiently to a diverse set of variant antigens). Therefore, by understanding how and which mutations increase flexibility and breadth, we may be able to make more informed decisions about which residues to protect or mutations to incorporate during the process of engineering Abs to have increased breadth, for example, against highly mutable pathogens.

## 2 Methods

### 2.1 Choice of antibodies to study

To understand the role of framework mutations on Ab structure and dynamics, we investigated the behavior of the fragment antigen-binding (Fab) region of five mature Abs (Atezolizumab, Daratumumab, Omalizumab, Pertuzumab, and Trastuzumab) both with and without GL-reverting mutations (herein, referred to as control Abs). This allowed us to parse out the effects of key single- and double-point mutations along the evolutionary trajectories of the clinically-relevant Abs of interest. Mutations are annotated throughout the text using the IMGT numbering scheme (e.g., X100Y, where X mutates to Y). The mutations were chosen based upon our previously-published (12) position specific scoring matrix (PSSM) for human Ab repertoires (**Table S1**), and were selected to include both high and low probabilities in the PSSM as well as a range of amino acid types.

### 2.2 Molecular dynamics simulations

Several new simulations were performed in this work and have been analyzed alongside simulations that we performed for a prior study (12). Here, we focused on applying various analysis techniques to elucidate novel Ab behaviors from this combined set of new and existing data. MD simulations were performed as described in our previous work (12). Briefly, the simulations consisted of an energy minimization step, equilibration steps in the NVT and then NPT ensemble, and finally a production simulation performed using the GROMACS (23) MD engine for 300 ns in the NPT ensemble with the Parrinello-Rahman barostat (24). While microseconds-long simulations are typically required to fully sample all Ab macrostates, as was recently shown by Fernández-Quintero et. al (10, 11)., here our goal is to assess the effects of key mutations on a single Ab macrostate. Based on our past work (12, 25) and the analysis described in the following section, we deemed 300 ns to be a sufficient amount of time for a single Ab macrostate to equilibrate and converge in solution.

### 2.3 Simulation trajectory analysis

We calculated the RMSD of the Abs over the course of the entire MD simulations. This allowed us to determine whether the simulations had converged (i.e., whether the RMSD had ceased to change noticeably over time), and to determine the number of stable Ab macrostates sampled within the simulated timescales. As a special case, the control simulation for Pertuzumab was cut short at 276 ns in subsequent analysis steps due to the large conformational change the Ab was observed to undergo at this time point, which resulted in a shift in the relative orientation between the variable and constant regions of the Ab towards a different macrostate.

We then calculated the RMSF of the Abs over the last 150 ns of each simulation to determine the per-residue flexibility of the Ab. Since all simulations were deemed to have converged by 150 ns, only the last 150 ns of each simulation were used to compute the RMSF. The RMSF profile of the control Ab was subtracted from that of each mutant Ab to determine how much the flexibility changed as a function of the mutation(s). The residues comprising each CDR loop were identified through an IgBlast (26) alignment or visual inspection of the antibody. Finally, we tabulated the solvent accessible surface area (SASA) to determine how the mutations changed the Ab geometry and packing of the *V_H_-V_L_* interface. A “shift” was calculated by subtracting the SASA of the control Ab from the mutant.

### 2.4 *V_H_-V_L_* interface angle analysis using ABangle

Using ABangle (27), we calculated the *V_H_-V_L_* interface angle every 100 frames for each of our simulations (approximately 150 calculations per simulation), leading to a distribution of *V_H_-V_L_* interface angles for each Ab. Additionally, we performed a two-tailed t-test on the angle distributions to quantify how different the angle distributions of the mutant Abs were from that of the control Ab (*=p-value< 0.05; **=p-value< 0.01; ***= p-value< 0.0001). These distributions were also fit to a combination of Gaussians and normalized. Finally, we calculated the average RMSD and mean *V_H_-V_L_* interface angle for each Ab over the last 150 ns of each simulation. We then subtracted the results for the control Ab from the mutant Abs to determine how much the mutant Abs “shift” in each of these metrics. After plotting these values, we found correlations by performing a linear regression.

## 3 Results

### 3.1 Crystallization in the presence of a bound ligand places constraints on antibody *V_H_-V_L_* interface angles

**Figure 1:**
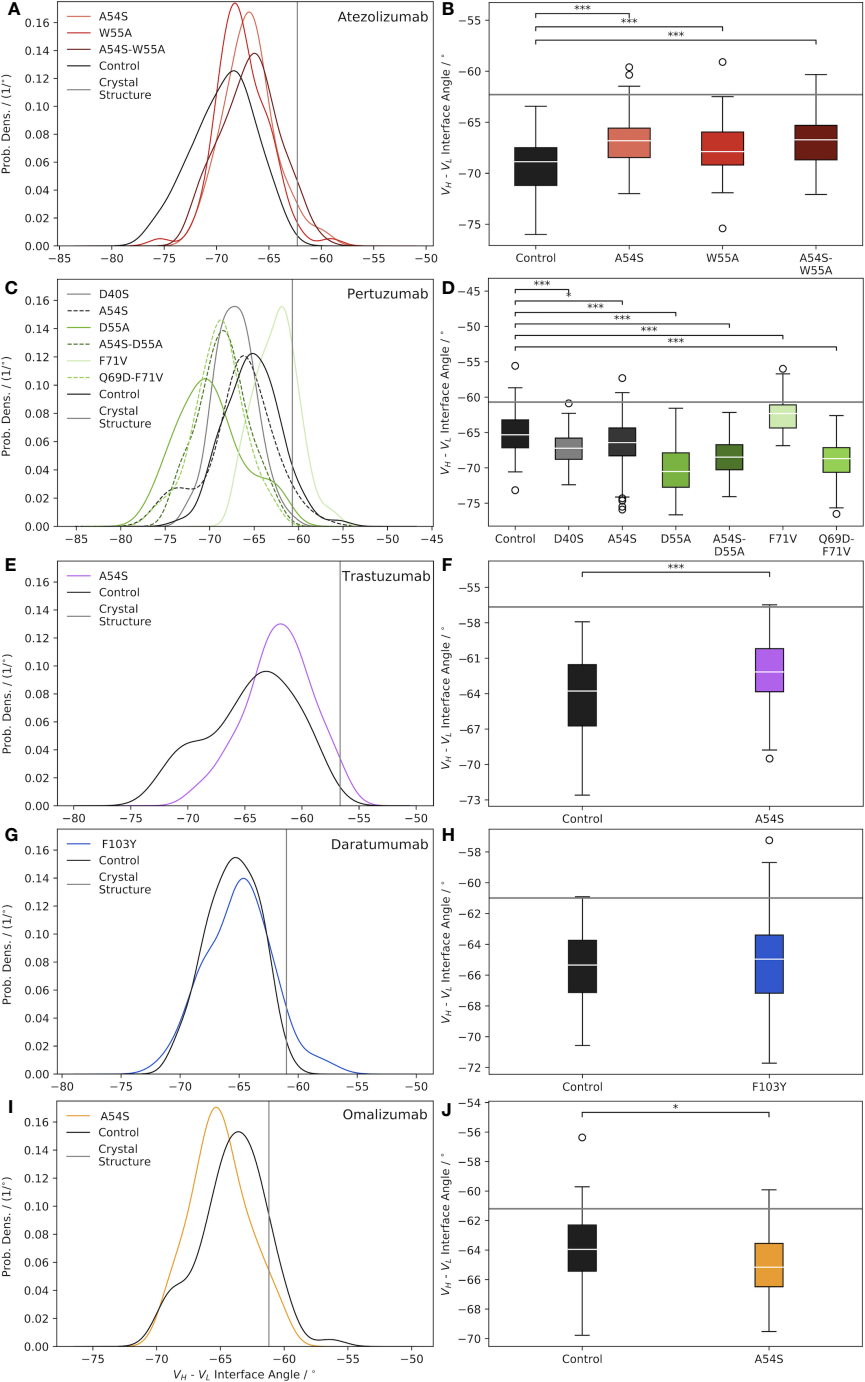
Characterization of Ab *V_H_-V_L_* interface angle distributions, shown as normalized probability densities (left plots) and boxplots (right plots), for: **(A, B)** Atezolizumab, **(C, D)** Pertuzumab, **(E, F)** Trastuzumab, **(G, H)** Daratumumab, and **(I, J)** Omalizumab. In each plot, a gray line indicates the Ab interface angle value for the respective crystal structure, and black curves/boxplots show the results for the mature (“Control”) Abs. Significance was assessed *via* a two-tailed t-test (*=p-value< 0.05; ***= p-value< 0.0001).

**Figure 1** illustrates how the *V_H_-V_L_* interface angles differ when comparing the crystal structures of the Abs to the ensemble of solution state Ab structures generated over the second half of the production MD simulations. For all five Abs we studied, we observe a shift towards more negative *V_H_-V_L_* interface angles for the ensemble of solution state structures (**Figure 1**, “control” distributions in black) compared to the crystal structures of the Abs (**Figure 1**, gray lines). These results are consistent with those of Fernández-Quintero et al. (10), who concluded that crystal structures, which represent single static snapshots, often cannot capture the high conformational diversity of correlated GL loop rearrangements and *V_H_-V_L_* interface orientations in solution. Here we observe that the same holds true for mature Abs.

How representative the *V_H_-V_L_* interface angle of the Ab crystal structure is of the ensemble of solution state structures appears, in part, to be dependent on whether or not a bound ligand is present in the crystal structure. For example, the crystal structures used in this work for Atezolizumab and Trastuzumab (PDB codes 5X8L (28) and 6B9Z (29), respectively) feature the Abs in complex with a ligand. Consequently, these two Abs have the largest differences between the crystal structure and solution state ensemble mean interface angle (*Δ*°_*Cryst* *Struc*−*Ensemble*_=7.1° for Trastuzumab and 6.6° for Atezolizumab; **Table S2**). In contrast, the crystal structures used for Daratumumab, Pertuzumab, and Omalizumab (PDB codes 7DUN, 4LLU (30), and 4X7S (31), respectively) feature unbound Abs, and exhibit correspondingly smaller differences between the crystal structure and ensemble mean interface angle values (*Δ*°_*Cryst* *Struc*−*Ensemble*_=4.3° for Daratumumab, 4.6° for Pertuzumab, and 2.7° for Omalizumab; **Table S2**).

A related finding to that from above was reported by Dunbar et al. (27), who looked at differences in the structural variation of bound and unbound Abs in the context of ligand specificity, comparing Abs specific for protein versus hapten antigens. Fernández-Quintero et al. noted other factors that may also play a role in this observation, including constraints placed on the *V_H_-V_L_* interface angle due to the crystallization process itself, such as distortion of the crystal structure by crystal packing effects (10, 11). We note there does not appear to be a correlation between the *Δ*°_*Cryst* *Struc*−*Ensemble*_ values due to the GL origins of the Abs, as, for example, Trastuzumab and Omalizumab are both V^H^3-66-class Abs but show very different behavior in this regard.

### 3.2 Introducing sequence mutations into mature Abs causes significant shifts in the *V_H_-V_L_* interface angle

**Figure 1A** illustrates the effects of reverting key framework mutations in Atezolizumab back to their GL identity on the relative interfacial orientation of the Ab heavy and light chain. Upon introducing the GL-reverting mutations A54S and W55A into the sequence of the mature Ab, the mean interface angles of the solution state ensemble distributions are found to shift to the right (versus the control/mature Ab distribution), towards the crystal structure value. Interestingly, there appears to be some degree of synergism in terms of how much the interface angle distribution shifts to the right upon mutating both residues simultaneously versus individually. **Figure 1B** shows that all the interface angle distribution shifts due to the single- and double-point mutations (e.g., one and two amino acid mutations) compared to the mature Ab, are significant.

Similar to the case for Atezolizumab, introducing key framework mutations into Pertuzumab causes significant shifts in the *V_H_-V_L_* interface angle, compared to the mature Ab (**Figures 1C, D**). In the case of Pertuzumab, we observe interface angle shifts both to the right *and* to the left of the control. Furthermore, where the W55A mutation in Atezolizumab led to a right-shift towards a less negative mean interface angle, the D55A mutation in Pertuzumab results in a dramatic shift to the left towards a more negative mean interface angle. Since Atezolizumab and Pertuzumab are from the same GL IGHV3-23 gene, this difference may be due to differences in mutation chemistry (e.g., W55A is an aromatic mutation vs. D55A is an acidic mutation).

Introducing the A54S GL-reverting mutation into Trastuzumab leads to a significant right-shift in the ensemble mean interface angle compared to the control (**Figures 1E, F**), as with Atezolizumab. For Daratumumab, we observe that introducing the F103Y mutation into the mature Ab sequence does not result in a significant shift in the mean interface angle (**Figures 1G, H**). Similarly, introducing the A54S mutation into the sequence of Omalizumab leads to only a minimally significant left-shift in the distribution towards more negative interface values (**Figures 1I, J**).

Intriguingly, across nearly all the Abs and mutations studied (4/5 Abs and 9/12 total mutations), we observe that compared to the control Abs, the mutated Abs sample a narrower distribution of interface angles (**Table S2**). While the reason for this observation remains unclear, we believe it is unlikely to be a sampling limitation upon introducing mutations into the Ab crystal structures, since we observe convergence in the range of interface angles explored during the simulations (**Figure S1**). Overall, these results highlight the need to account for interface angle changes when engineering mutations into Ab sequences, since, as we discuss next, these changes can have important consequences for Ab conformational stability, flexibility, and possibly even binding.

### 3.3 Shifts in the *V_H_-V_L_* interface angle from the control Ab correlate with changes in RMSD, but have little impact on Ab conformational stability

**Figure 2:**
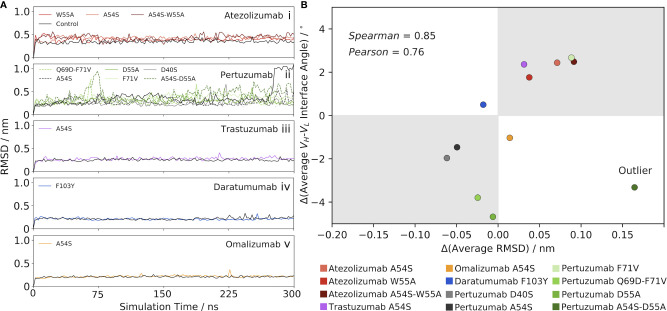
Mutation-induced shifts in the *V_H_-V_L_* interface angle away from the control Ab correlate with changes in RMSD from the control. **(A)** RMSD as a function of simulation time for (i) Atezolizumab, (ii) Pertuzumab, (iii) Trastuzumab, (iv) Daratumumab, and (v) Omalizumab, and their mutants. **(B)** Correlation between the change in *V_H_-V_L_* interface angle (mutant – control) and change in RMSD (mutant – control). One outlier was identified *via* an outlier analysis (see main text) and excluded from the calculation of the reported correlation values.

We anticipated that changes in the *V_H_-V_L_* interface angle upon mutation might impact the conformational stability and/or flexibility of the Abs. To this end, we calculated the RMSD of each Ab over the course of the production MD simulation, in reference to its respective starting structure, which is essentially the same as the crystal structure (and so will be referred to as such) since restraints are removed only at the beginning of each production run. **Figure 2Ai** illustrates the results for Atezolizumab, which show a higher average RMSD for the mutant Abs with the A54S and/or W55A mutations than for the control Ab. Moreover, we observe a higher average RMSD for the double-point mutant than for the single-point mutants.

The RMSD results for Atezolizumab contrast those of **Figure 1A**, which show the mean interface angle for the control Ab shifted *furthest* from the crystal structure value, and that of the double-point mutant shifted the *least* from the crystal structure value, with the values for the single-point mutants falling in-between. If changes in the *V_H_-V_L_* interface angle are driving the observed changes in RMSD, we would expect to see the mutants have a *lower* overall RMSD than the control, as they prefer an interface orientation similar to that of the crystal structure; however, our findings show the opposite is true. In other words, the increased RMSD of the mutants above the control Ab do not appear to be a direct result of the greater *V_H_-V_L_* orientational shifts of the mutants from the reference (crystal) structure used in the RMSD calculations, but are instead a product of a cascade of effects. More specifically, we hypothesized that the mutation-induced changes in the interface angles instead mediate changes in the *flexibility* of the Abs, which is the key driving force behind the observed increases in RMSD of the mutant Abs above the control. Feeding into this hypothesis, the RMSD is seen to stabilize quickly in each simulation, indicating the overall conformational stability of the Abs is relatively unaffected by the changes in the interface angles. We explore this flexibility hypothesis in detail in the following section.

Since the sign of the change in interface angle is clearly highly dependent on the reference (crystal) structure, we aimed to eliminate this bias by only comparing the mutant Ab data to the control Ab data, rather than to the crystal structure. RMSD stabilizes quickly in each simulation, indicating the interface angle shifts upon mutation have little impact on the conformational stability of the Abs. This led us to look at the results for Atezolizumab from this perspective, we observe that greater interface angle shifts to the right of the control consistently lead to greater increases in RMSD above the control (**Figures 1A**, **2Ai**). Similarly, introducing the F71V mutation into Pertuzumab or the A54S mutation into Trastuzumab leads to a right-shift in the mean interface angle and a higher RMSD compared to the control (Pertuzumab: **Figures 1C**, **2Aii**, Trastuzumab **Figures 1E**, **2Aiii**). Vice versa, introducing the D40S, A54S, D55A, and Q69D-F71V mutations into Pertuzumab leads to a left-shift in the mean interface angle and a *lower* RMSD compared to the control. For Daratumumab (**Figures 1G**, **2Aiv**) and Omalizumab (**Figures 1I**, **2Av**), the results show negligible or only minor changes in RMSD between the mutant and control, and, correspondingly, little-to-no shifts in the interface angle.

To better quantify this trend across all five of the Abs, we plotted the average change in RMSD versus the average change in the *V_H_-V_L_* interface angle upon mutation (**Figure 2B**), using the control Ab as the reference. Excluding one outlier—the A54S-D55A mutation in Pertuzumab, identified *via* an influence analysis to have a large residual and high leverage on the linear regression (**Figure S2B**)—the data shows a strong correlation exists between these variables (Pearson correlation coefficient = 0.76 and Spearman’s Rank correlation coefficient = 0.85).

### 3.4 Increases in solvent accessible surface area lead to increases in flexibility, triggered by *V_H_-V_L_* interface angle shifts

**Figure 3:**
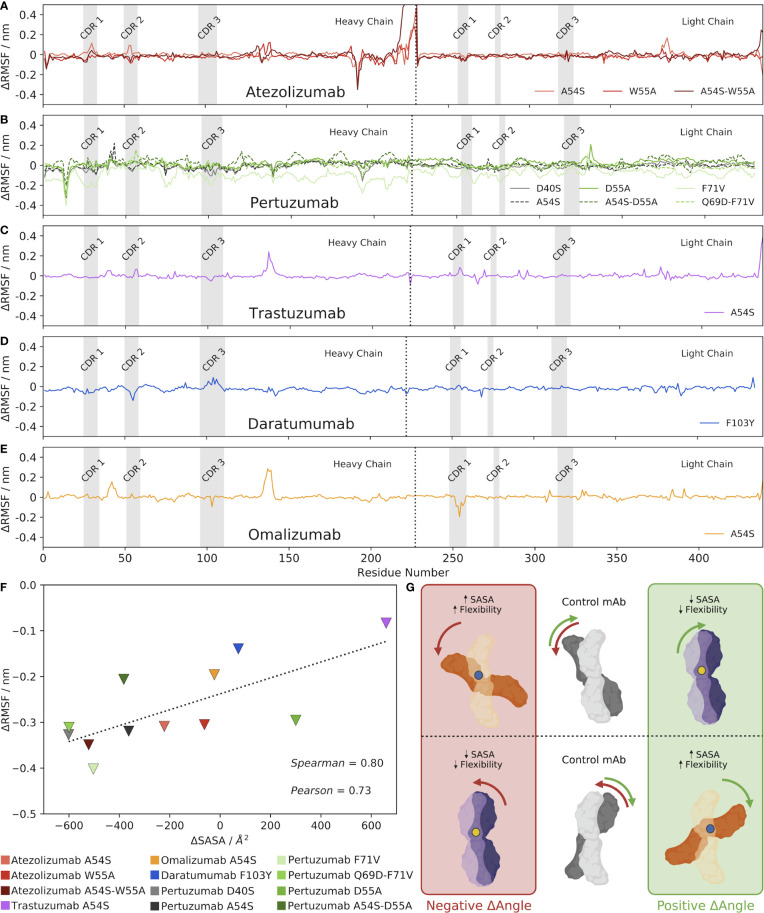
Mutations alter the *V_H_-V_L_* interface angle, altering the SASA of the Ab, which alters its overall flexibility. **(A–E)** Changes in RMSF between each mutant Ab and the control, mature Ab. **(F)** Correlation between changes in Ab SASA and RMSD upon mutation, compared to the control Ab. Triangles depict the minimum changes in RMSF between the mutant and control Ab as the change in SASA shifts, with a dashed line included simply for ease of visualization. **(G)** Visual hypothesis of these results. The middle panel shows two example control states where the mature Ab is oriented with a specific *V_H_-V_L_* interface angle. When a mutation is made, shown as either a blue or yellow dot, the *V_H_-V_L_* interface angle shifts. However, a positive change in *V_H_-V_L_* interface angle does not always indicate an increase in SASA. As shown in the diagram, an increase in SASA (and subsequently flexibility) is a result of a blue mutation shown in the orange Abs. Conversely, a decrease in SASA (and subsequently flexibility) is a result of a yellow mutation shown in the purple Abs.

To rigorously explore the potential relationship between changes in the *V_H_-V_L_* interface angle, RMSD, and Ab flexibility upon mutation, we computed the per-residue RMSF of each Ab over the course of the production MD simulation. We then subtracted the RMSF value for the control Ab at each residue, the results of which are shown in **Figures 3A–E** for the five Abs and their mutants. Analyzing the results for the Atezolizumab mutants first (**Figure 3A**), we were surprised to see little-to-no change in fluctuation at the sites of the actual mutations (positions 54 and 55). Instead, we observe both positive and negative fluctuations at several other sites along the sequence of the Abs, which often appear to overlap for the three mutant Abs (e.g., around positions 164, 215, 348, and 406). We observe similar trends when analyzing the RMSF profiles of the other Abs as well, in that the greatest changes in RMSF do not typically occur near the actual mutation site(s). Notably, for Daratumumab, where no significant change was observed in the *V_H_-V_L_* interface angle or RMSD with the F103Y mutation, we also see fewer and more subtle changes in RMSF compared to the control (**Figure 3D**), relative to the other Abs/mutations we studied.

From these results, we hypothesized that mutation-induced shifts in the *V_H_-V_L_* interface angle led to changes in the contact area between the Ab heavy and light chains, causing changes in the overall SASA of the Abs. More precisely, we expect that observed *increases* in RMSF/flexibility upon a mutation-induced *V_H_-V_L_* angle shift might be specifically occurring at Ab regions that experience *increased* SASA compared to the control, and vice versa, observed *decreases* in flexibility might be occurring at Ab regions of *decreased* SASA. In other words, residues that lose amino acid contacts and are more solvent-exposed after the *V_H_-V_L_* angle shift should experience increased flexibility, whereas residues that gain amino acid contacts and are less solvent-exposed after the angle shift (have less space to maneuver) should experience decreased flexibility.

To explore this idea, we calculated the minimum and maximum difference in RMSF between each mutant and its respective control Ab, ignoring the first and last 10 residues of the Abs that are known to be highly flexible and could skew our results. We decided to look specifically at the largest differences in RMSF–both positive and negative— between the mutant and the control because comparing the average RMSF values would likely dampen any overall trends by cancelling out negative and positive fluctuation changes. We also calculated the SASA of each Ab, and then plotted these values separately against the minimum and maximum RMSF changes for each Ab (**Figures 3F**, **S3**, respectively). The results show a clear correlation exists between the minimum change in RMSF and the change in SASA upon mutation (Pearson correlation coefficient = 0.73 and Spearman’s Rank correlation coefficient = 0.80). Interestingly, a much weaker correlation exists between the maximum change in RMSF and the change in SASA upon mutation (Pearson correlation coefficient = 0.55 and Spearman’s Rank correlation coefficient = 0.59).

We emphasize that while many of the mutations induced a clear increase or decrease in the overall SASA of the Ab compared to the control, the RMSF profiles of these mutant Abs still generally showed both positive *and* negative fluctuations in RMSF; we simply observe fewer and/or smaller positive fluctuations for mutants that increased the overall SASA of the Ab, and vice versa. **Figure S4** shows a visual depiction of how a given change in the *V_H_-V_L_* interface angle can result in both increases and decreases in flexibility at different locations in the Ab structure. In addition, **Figures S5**-**S9** show molecular representations from the simulation trajectories of the Ab residues that experience the maximum and minimum changes in flexibility/RMSF compared to the control, which support the notion that these changes generally occur in areas that experience changes in SASA as a result of the change in interface angle upon mutation.

Given, as was mentioned earlier, that the orientation and packing of the *V_H_-V_L_* interface has been shown to affect the orientation of CDR loops (32), we sought to identify whether changes in flexibility/RMSF might be specifically occurring in the CDRs, which are frequently found exposed to solvent. To this end, we determined the residues comprising the heavy and light chain CDR regions of each Ab (see Methods), which are shown as gray regions in **Figures 3A–E**. The results show that in some cases, notable changes in RMSF do sometimes occur in the CDR regions, including in the CDR1 and CDR2 of Atezolizumab’s heavy chain with the A54S mutation (**Figure 3A**), the CDR2 and CDR3 of Daratumumab’s heavy chain with the F103Y mutation (**Figure 3D**), and the CDR 1 of Omalizumab’s light chain with the A54S mutation. Yet, more often than not, the minimum and maximum changes in RMSF upon mutation do *not* occur in the CDR regions. This is not entirely surprising, given the CDRs may not be the Ab regions experiencing the greatest increases or decreases in SASA upon mutation.

Overall, these results provide strong evidence for our hypothesis, which is visually described in **Figure 3G**, wherein changes in the *V_H_-V_L_* interface angle upon mutation lead to changes in the overall SASA of the Ab, which in turns increases the localized flexibility/RMSF of the residues that directly experience these changes in SASA. **Figure 3G** also highlights the idea that the reference structure, and how the antibody shifts in comparison to it as a result of a mutation, can influence the sign of the ensuing SASA change with respect to the changing *V_H_-V_L_* interface angle.

## 4 Conclusion and discussion

Fernández-Quintero et. al (10, 11). analyzed the *V_H_-V_L_* interface of 16 different combinations of GL Abs and found that the structures may be more accurately described as “conformational ensembles” in solution. Additionally, the *V_H_-V_L_* dynamics appear to occur on a low nanosecond timescale. Therefore, we decided to take a second look at several framework mutations we previously explored, to better understand the role of *V_H_-V_L_* dynamics in mature Abs.

We began by translating the analysis done by Fernández-Quintero et al. to mature, FDA approved Abs and found that, similar to Fernández-Quintero et. al., the crystal structure indeed does not represent the conformational ensemble of the Ab; the torsion angle describing the *V_H_-V_L_* is observed to significantly shift when the structures are allowed to equilibrate in an aqueous solution. Additionally, we found that many of the GL-reverting mutations we studied resulted in narrower conformational distributions, implying an overall rigidification of the Abs. At first glance, this result appears to challenge the accepted paradigm that GL antibodies evolved to be flexible in order to be capable of binding to a wide array of antigens (16). A similar result was also reported by Ovchinnikov et al., wherein GL B cells that have a high affinity for conserved antigenic residues at the start of affinity maturation, will acquire framework mutations that increase antibody rigidity over time (16). Importantly, however, many of the mutations we studied were still found to mediate *local* changes in antibody flexibility, discussed in more detail below.

Before we can utilize the interface orientation as a parameter, we first must better understand what influences the *V_H_-V_L_* interface angle and sidechain packing in a variety of conditions. One important aspect which warrants additional analysis is the role of ligand binding on the interfacial orientation. The data presented in this case study suggest that the presence of a ligand causes the *V_H_-V_L_* interface to shift. These implications could vary depending on the type of ligand that is co-crystallized (protein, hapten, peptide, etc.) with the Ab, as has been noted in the literature (27).

Many crystal structures deposited in the Protein Data Bank (PDB) are co-crystallized with a ligand. The presence of a ligand can aid in resolving a high-quality crystal structure and reveal critical information about the paratope of the Ab under study, however, it also places structural restraints on the Ab of interest. The impact of these restraints can, of course, be biophysically relevant for the integrity of the complex, but the restraints can also bias our understanding of how the Ab will behave in solution before it meets its cognate antigen.

Next, we found that framework mutations can significantly impact the *V_H_-V_L_* interface angle. While not all mutations studied had a significant impact on the *V_H_-V_L_* interface angle, or had the same impact across different Abs, we did see that even mutations distant from the *V_H_-V_L_* interface could impact the relative orientation of the Ab heavy and light chain. These findings highlight the need to more broadly study the effects of mutations on the *V_H_-V_L_* interface angle, in order to use this parameter as a tool to tune and develop Abs with desired properties.

Furthermore, we found that the Ab mutations that impacted the *V_H_-V_L_* interface angle also had correlated shifts in their RMSD values, but that these shifts were not due to changes in the overall conformational stability of the Abs, nor simply due to geometric changes in the antibody away from the reference (crystal) structure. This, in turn, led us to investigate the impact of the framework mutations on the SASA and RMSF of the Abs.

We found that when a mutation increases the overall SASA of the Ab, it results in an increase in the flexibility of the newly solvent-exposed regions of the Ab (and vice versa). We believe this is due to the Ab having fewer constraints in a less tightly-packed conformation. Our hypothesis is that increases in SASA are directly due to changes in the *V_H_-V_L_* interface angle, as these shifts expose more of the Ab to solvent and decrease the contact angle between the Ab heavy and light chains. However, because the *V_H_-V_L_* interface angle can shift either right or left to increase the SASA, it is difficult to discern if this is truly causative and more studies need to be done to fully validate this hypothesis. We also note that with mutations that increase the overall SASA of the Ab, other regions of the Ab may still experience a slight *decrease* in SASA and thus flexibility due to the angle shift, evidenced by both positive and negative fluctuations (compared to the control) in the RMSF profiles. The reverse holds true for mutations that decrease the overall SASA.

These findings may have important implications for understanding antibody mechanisms of action and for rational Ab design and offers another parameter to tune when engineering potential Ab therapies. It is clear from our and others’ work that the *V_H_-V_L_* interface angle can vary in solution, but the reasons behind these shifts have yet to be fully characterized and understood, which is necessary to realize any potential benefits.

As we seek to tackle increasingly difficult issues facing humanity—from diseases to separations—more sophisticated tools must be developed to engineer novel Ab formulations. The broad range of interfacial orientations observed in GL Abs suggests this parameter could play an important role in our natural immune defense systems. Indeed, the orientation and packing of the *V_H_-V_L_* interface has long been shown to influence the orientation of the CDR loops, and in turn, the antigen specificity profile (32).

As molecular algorithms and machine learning approaches rapidly advance through sequence/structure-to-function predictions, it will also be important to develop our knowledge of how the training sets may bias Ab engineering efforts. Crucially, many of these models are trained to predict the static protein crystal structure. As we have seen from this case study and the work of Fernández-Quintero et al. (10, 11), these crystal structures may not accurately capture the ensemble of solution-state structures of the Ab arising from the dynamics of the system. Advances in our understanding of the protein crystallization process through both computational and experimental approaches could reveal solution-to-crystal mechanistic rules, and in turn, allow us to accurately predict the solution conformational ensemble without the need for extensive molecular simulations and enhanced sampling approaches. Recent advances published from the Baker group demonstrate the ability to computationally design the 3D crystal lattice of proteins (33), and serves as a step forward in our understanding of the rules governing protein interactions during the crystallization process.

Looking forward, our work suggests that mutations that shift the *V_H_-V_L_* interface angle, change the overall SASA of the Ab and mediate changes in localized Ab flexibility. Further, the data presented herein suggests this relationship is monotonic and positively correlated. The higher Spearman coefficient (relative to the Pearson coefficient) may indicate that this relationship is not necessarily linear; however, given how close the two coefficient values are, additional simulations are warranted to elucidate the exact nature of the correlations. The ability to precisely tune Ab flexibility could contribute to increasing the number of macrostates the Ab visits, allowing it to sample a wider range of unique conformations and thus bind to more diverse antigens and ligands. In addition, other novel real-world impacts of modulating Ab flexibility could become known through future experimental testing. In particular, prospective studies such as *in silico* mutagenesis could be performed to either increase or decrease flexibility and observe the effects on affinity maturation, etc., which could then be tested *via* experimental affinity and kinetics measurements. Current Ab engineering techniques tend to optimize solely for binding affinity; our results imply a new approach might be fruitful where in addition to seeking out strong binders, we also find Abs that utilize flexibility to overcome energy barriers.

## Data availability statement

The original contributions presented in the study are included in the article/**Supplementary Materials**. Further inquiries can be directed to the corresponding author.

## Author contributions

KS, ER, JF, and BP designed research, performed research, analyzed data, and wrote the paper. All authors contributed to the article and approved the submitted version.
