# Supplementary material for: Common framework mutations impact antibody interfacial dynamics and flexibility
Source: Front Immunol. 2023 Feb 23;14:1120582. doi: 10.3389/fimmu.2023.1120582 (PMC9996335; doi:10.3389/fimmu.2023.1120582)
Supplement: Supplementary file 1 [file DataSheet_1.pdf]

Supporting Information

Common Framework Mutations Impact Antibody Interfacial Dynamics and Flexibility

*Emily Rhodes, Jon Faris, Brian Petersen, and Kayla Sprenger**

Department of Chemical & Biological Engineering, University of Colorado Boulder, CO 80303

*corresponding author: kayla.sprenger@colorado.edu

Position Specific Scoring Matrix

Mutation	VH3-23	VH3-23 nt	VH3-66	VH3-66 nt
40D	6.1	2	8.4	2
54A	20.3	1	17.1	1
55D	4.6	0	2.6	1
69Q	1.6	2	-0.2	2
71F	-0.7	2	-2.4	2
103F	22.0	1	22.3	1

Table S1. Position specific scores for the mutations and germlines selected. These scores demonstrate the spectrum of mutation probability and type.

Antibody Name	Mutation	Median	Mean	Standard Deviation	Minimum	Maximum	Crystal Structure
Atezolizumab	A54S	-66.8	-66.9	2.48	-72.0	-59.6	-62.3
Atezolizumab	W55A	-67.9	-67.5	2.46	-75.4	-59.1	-62.3
Atezolizumab	A54S-W55A	-66.7	-66.8	2.73	-72.1	-60.3	-62.3
Atezolizumab	Control	-68.9	-69.3	2.95	-76.0	-63.4	-62.3
Daratumumab	F103Y	-65.0	-65.0	2.81	-71.7	-57.2	-61.0
Daratumumab	Control	-65.3	-65.5	2.19	-70.6	-60.9	-61.0
Omalizumab	A54S	-65.2	-65.0	2.27	-69.5	-59.9	-61.2
Omalizumab	Control	-63.9	-64.0	2.59	-69.8	-56.4	-61.2
Pertuzumab	D40S	-67.2	-67.2	2.31	-72.4	-60.9	-60.7
Pertuzumab	A54S	-66.4	-66.7	3.83	-75.9	-57.3	-60.7
Pertuzumab	D55A	-70.5	-69.9	3.78	-76.6	-61.6	-60.7
Pertuzumab	A54S-D55A	-68.5	-68.5	2.77	-74.1	-62.2	-60.7
Pertuzumab	F71V	-62.3	-62.5	2.39	-66.9	-56.0	-60.7
Pertuzumab	Q69D-F71V	-68.7	-69.0	2.84	-76.5	-62.6	-60.7
Pertuzumab	Control	-65.3	-65.2	3.09	-73.2	-55.6	-60.7
Trastuzumab	A54S	-62.2	-62.1	2.94	-69.5	-56.5	-56.7
Trastuzumab	Control	-63.8	-64.4	3.86	-72.6	-57.9	-56.7

Table S2. Properties of the V_H-V_L interface angle distribution for each antibody-mutation combination. The crystal structure V_H-V_L interface angle was calculated for each antibody and is included for reference.

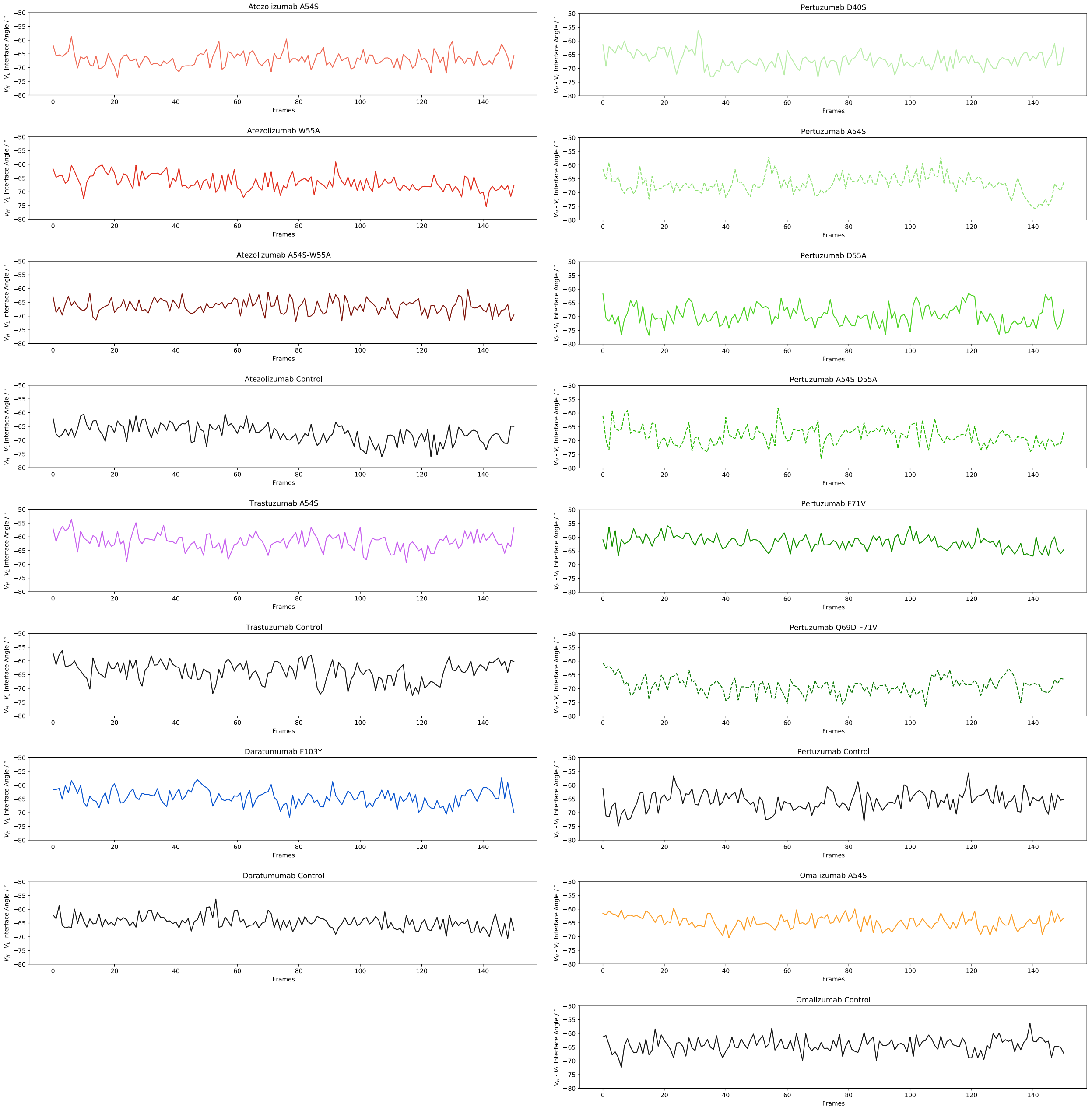

Figure S1. The V_H-V_L interface angle is plotted as a function of the frames taken from each simulation. A calculation of the V_H-V_L interface angle was done every 100 printed steps in the simulation, providing 150 data points per simulation. The second half of the trajectory (and subsequent frames) were used for calculating V_H-V_L interface angle distribution properties to ensure that the converged portion of the simulation is used for data analysis.

A

Antibody	Mutation	RMSD	Angle	Data Point Number
Atezolizumab	A54S	0.07	2.44	0
Atezolizumab	W55A	0.04	1.76	1
Atezolizumab	A54S-W55A	0.09	2.48	2
Pertuzumab	D40S	-0.06	-1.97	3
Pertuzumab	A54S	-0.05	-1.47	4
Pertuzumab	D55A	-0.01	-4.68	5
Pertuzumab	A54S-D55A	0.16	-3.32	6
Pertuzumab	F71V	0.09	2.67	7
Pertuzumab	Q69D-F71V	-0.02	-3.80	8
Daratumumab	F103Y	-0.02	0.50	9
Omalizumab	A54S	0.01	-1.03	10
Trastuzumab	A54S	0.03	2.36	11

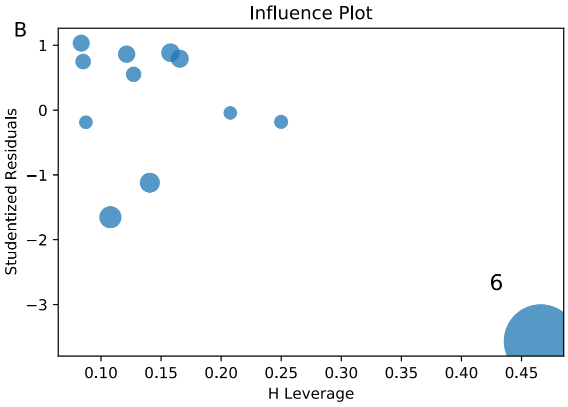

Figure S2. (A) A table denoting the points displayed in the influence plot. (B) An influence plot indicating that point 6, Pertuzumab A54S-D55A, has both higher leverage and heavier studentized residuals than all the other points included in the analysis. As a result of this analysis, point 6 was left out of the regression analysis between change in RMSD and change in V_H-V_L interface angle.

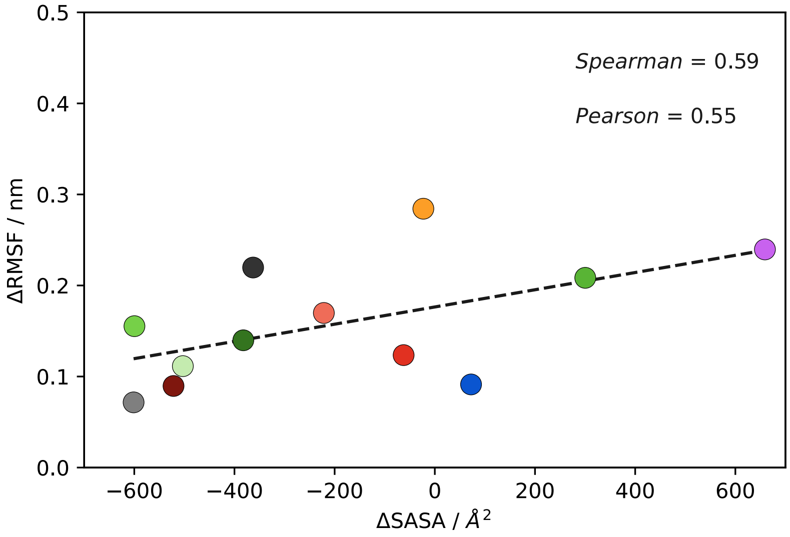

Figure S3. Correlation between changes in Ab SASA and RMSD upon mutation, compared to the control Ab. Circles depict the maximum changes in RMSF between the mutant and control Ab as the change in SASA shifts, with a dashed line included simply for ease of visualization.

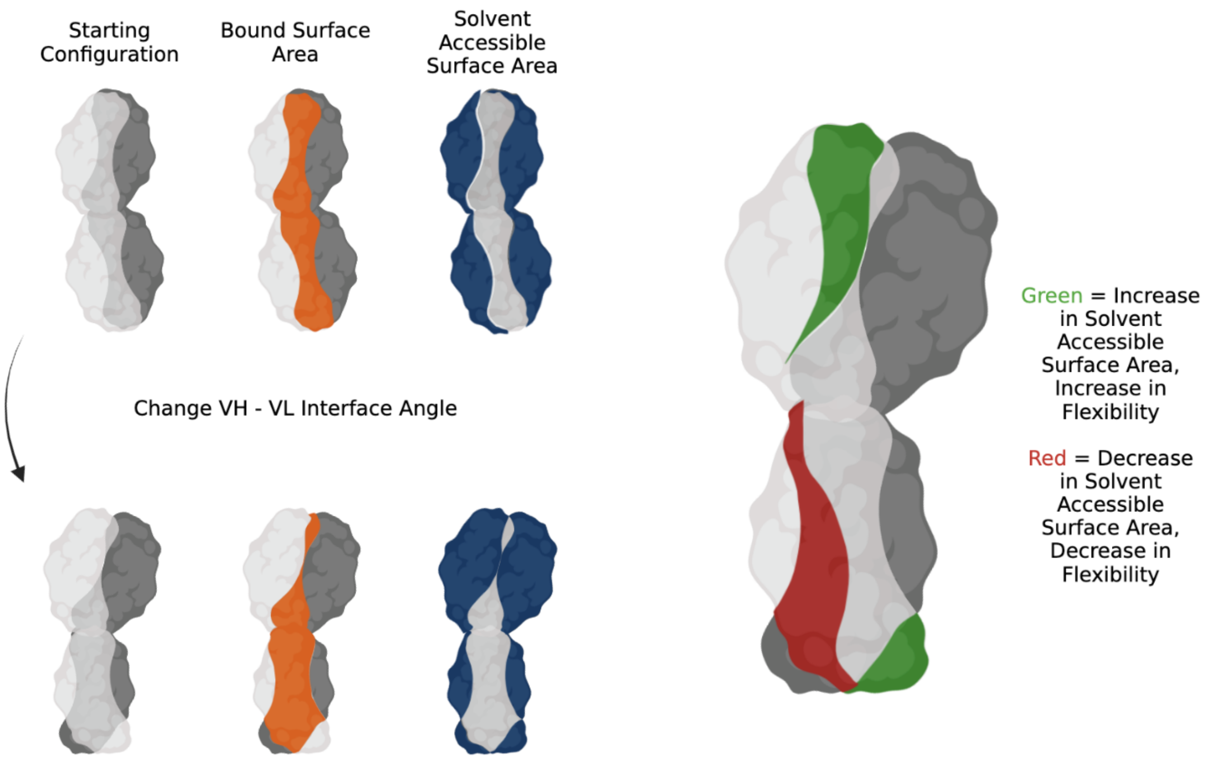

Figure S4. A change in V_H – V_L interface angle results in changes in solvent accessible surface area in a variety of locations within the antibody, that subsequently impact antibody flexibility.

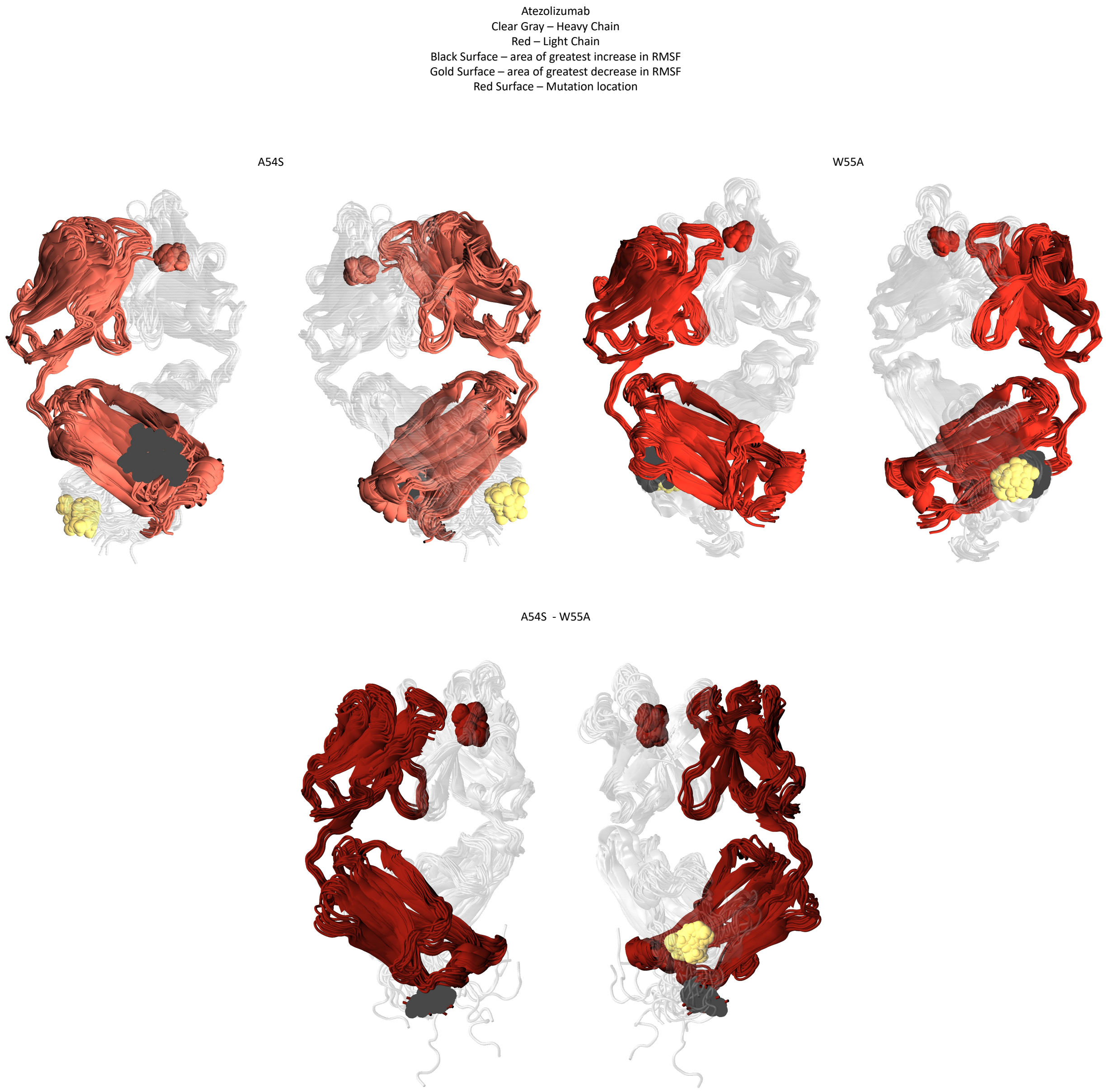

Figure S5. Visualization of the areas of with the largest increase and decrease of flexibility for Atezolizumab.

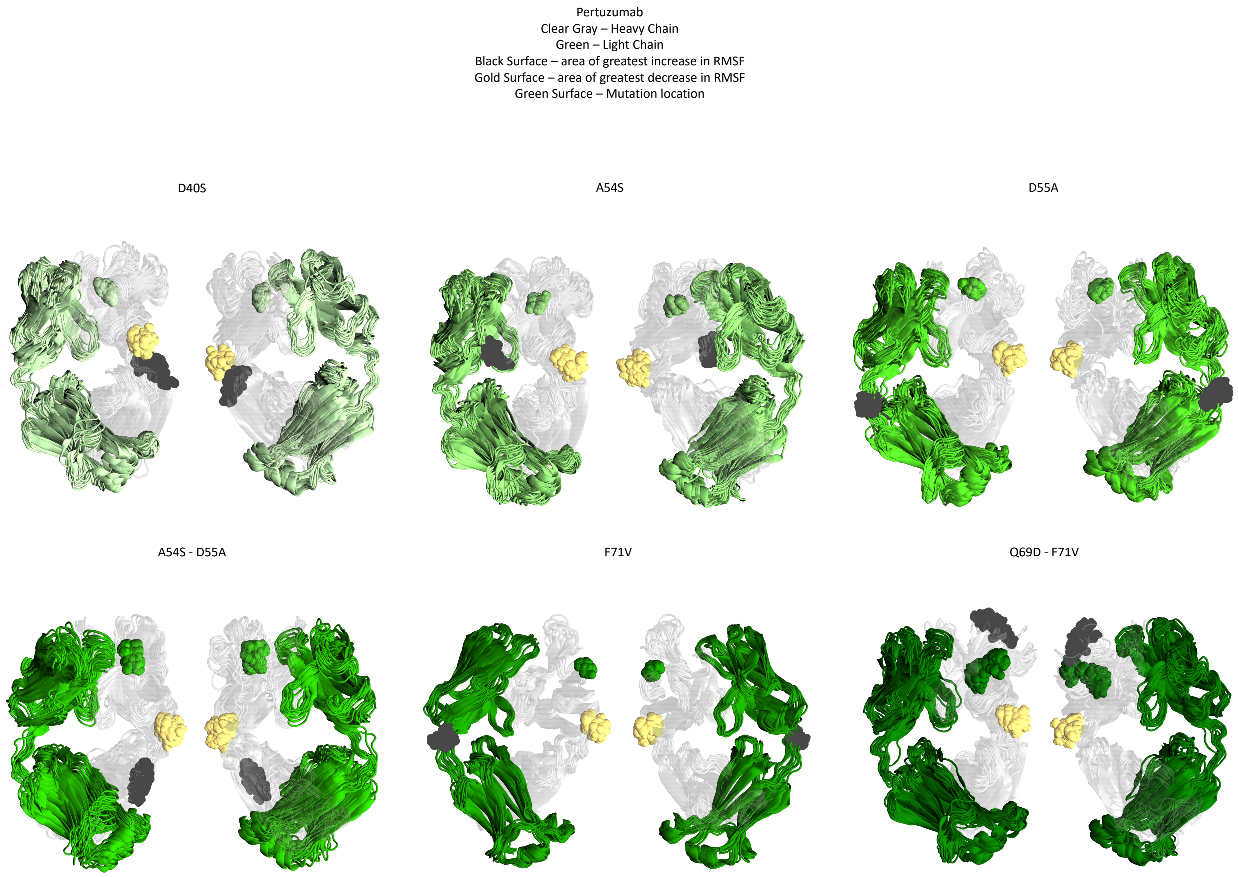

Figure S6. Visualization of the areas of with the largest increase and decrease of flexibility for Pertuzumab.

Daratumumab
Clear Gray – Heavy Chain
Blue – Light Chain
Black Surface – area of greatest increase in RMSF
Gold Surface – area of greatest decrease in RMSF
Blue Surface – Mutation location

F103Y

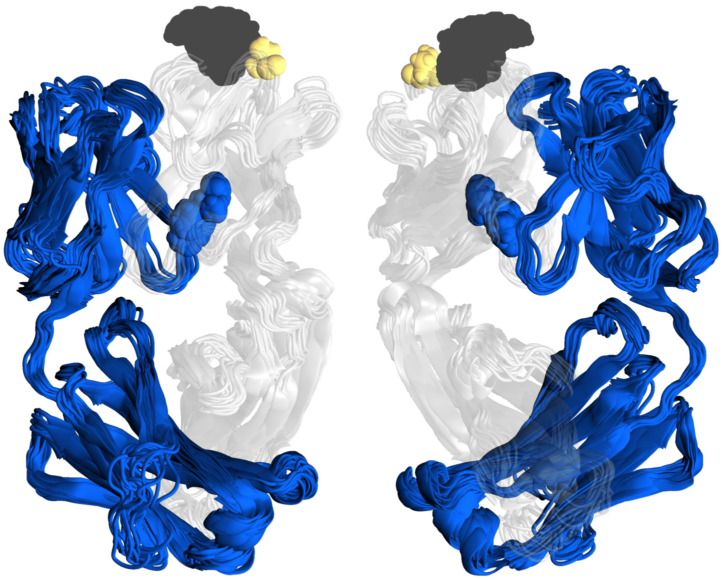

Figure S7. Visualization of the areas of with the largest increase and decrease of flexibility for Daratumumab.

Omalizumab
Clear Gray – Heavy Chain
Orange – Light Chain
Black Surface – area of greatest increase in RMSF
Gold Surface – area of greatest decrease in RMSF
Orange Surface – Mutation location

A54S

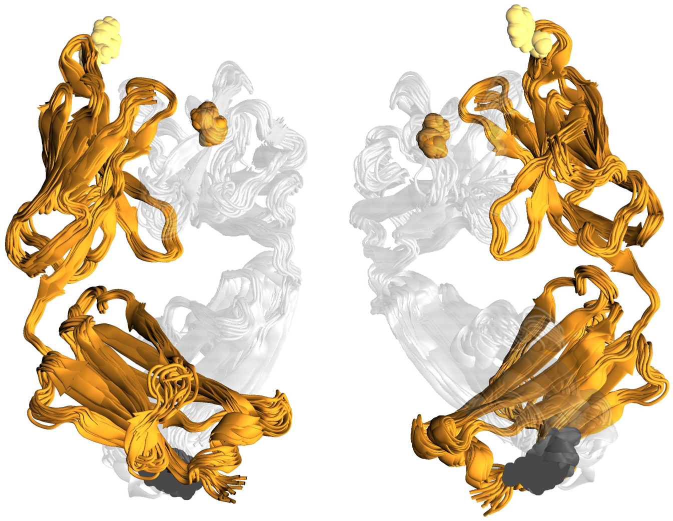

Figure S8. Visualization of the areas of with the largest increase and decrease of flexibility for Omalizumab.

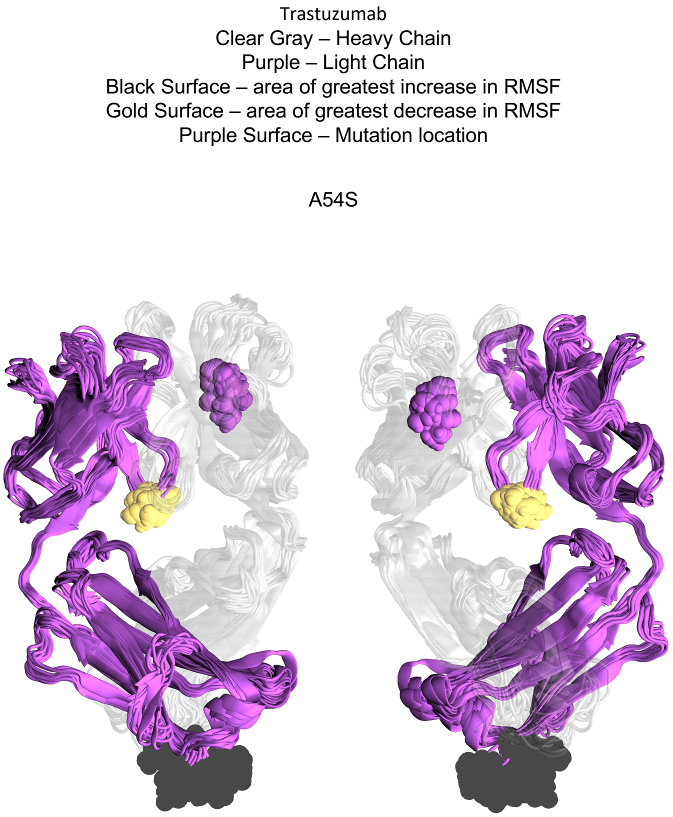

Figure S9. Visualization of the areas of with the largest increase and decrease of flexibility for Trastuzumab.
